# Individual differences in musical ability among adults with no music training

**DOI:** 10.1177/17470218221128557

**Published:** 2022-10-27

**Authors:** Ana Isabel Correia, Margherita Vincenzi, Patrícia Vanzella, Ana P Pinheiro, E Glenn Schellenberg, César F Lima

**Affiliations:** 1 Centro de Investigação e Intervenção Social (CIS-IUL), Instituto Universitário de Lisboa (ISCTE-IUL), Lisboa, Portugal; 2 Department of General Psychology, University of Padova, Padova, Italy; 3 Center for Mathematics, Computing and Cognition, Universidade Federal do ABC, Santo Andre, Brazil; 4 CICPSI, Faculdade de Psicologia, Universidade de Lisboa, Lisbon, Portugal; 5 Department of Psychology, University of Toronto Mississauga, Mississauga, ON, Canada; 6 Institute of Cognitive Neuroscience, University College London, London, UK

**Keywords:** Music, ability, training, cognition, personality

## Abstract

Good musical abilities are typically considered to be a consequence of music training, such that they are studied in samples of formally trained individuals. Here, we asked what predicts musical abilities in the absence of music training. Participants with no formal music training (*N* = 190) completed the Goldsmiths Musical Sophistication Index, measures of personality and cognitive ability, and the Musical Ear Test (MET). The MET is an objective test of musical abilities that provides a Total score and separate scores for its two subtests (Melody and Rhythm), which require listeners to determine whether standard and comparison auditory sequences are identical. MET scores had no associations with personality traits. They correlated positively, however, with informal musical experience and cognitive abilities. Informal musical experience was a better predictor of Melody than of Rhythm scores. Some participants (12%) had Total scores higher than the mean from a sample of musically trained individuals (⩾6 years of formal training), tested previously by Correia et al. Untrained participants with particularly good musical abilities (top 25%, *n* = 51) scored higher than trained participants on the Rhythm subtest and similarly on the Melody subtest. High-ability untrained participants were also similar to trained ones in cognitive ability, but lower in the personality trait openness-to-experience. These results imply that formal music training is not required to achieve musician-like performance on tests of musical and cognitive abilities. They also suggest that informal music practice and music-related predispositions should be considered in studies of musical expertise.

Musical abilities and behaviours vary widely across individuals. Some people do not value music and struggle with music-related activities (e.g., singing in tune, dancing in time), whereas others have sophisticated musical skills and display a diverse repertoire of musical behaviours. In the scientific literature and in Western societies, good musical abilities tend to be equated with formal training and being proficient at singing or playing a musical instrument (e.g., Ullén et al., 2014; Wallentin et al., 2010).

Accordingly, most of the relevant literature has compared groups of formally trained individuals to those with no training, so-called *nonmusicians*, whether the design is cross-sectional (e.g., Lima & Castro, 2011; MacDonald & Wilbiks, 2021; Schellenberg & Mankarious, 2012; Tierney et al., 2020) or longitudinal (e.g., Martins et al., 2018; Roden et al., 2014; Schellenberg et al., 2015; Thompson et al., 2004). Findings from these studies inform debates about associations between music lessons and nonmusical abilities (e.g., speech perception, executive functions). Although transfer effects of music training remain the focus of much debate (e.g., Bigand & Tillmann, 2022; Degé, 2021; Kragness et al., 2021; Martins et al., 2021; Sala & Gobet, 2020; Schellenberg, 2020), learning to play an instrument involves honing several cognitive skills, such as attention, memory, and self-discipline (Wan & Schlaug, 2010). Music lessons might therefore have relevant implications for education, health, and well-being.

Because researchers are typically interested in possible side-effects of formal music training (i.e., plasticity or transfer), even when causation cannot be inferred (see Schellenberg, 2020), untrained individuals tend to be treated as a homogeneous group regarding their musicality, or musical ability. The presumption is that untrained individuals have poor musical abilities, such that music training and musical abilities are conflated. The fact that many studies of associations between music training and nonmusical abilities do *not* measure musical abilities confirms that musicality is thought to be high in the trained group and low in the untrained one.

Recent findings raise doubts about this assumption. First, an established genetic component to musical ability and achievements means that natural variation in musical abilities is expected even in the absence of training (Gingras et al., 2015; Mosing et al., 2014; Tan et al., 2014). Second, when music training is held constant, individuals with good musical ability show enhanced nonmusical skills including speech processing (Mankel & Bidelman, 2018; Swaminathan & Schellenberg, 2017) and vocal emotion recognition (Correia et al., 2022a), mirroring the enhancements seen in formally trained musicians. Indeed, when music training and musical ability are considered jointly, associations between training and nonmusical abilities often disappear (Correia et al., 2022a; Swaminathan & Schellenberg, 2020; Swaminathan et al., 2017, 2018). Third, some musical capacities are achieved simply by engaging in music-related activities, such as listening to music (e.g., Bigand & Poulin-Charronnat, 2006; Larrouy-Maestri et al., 2017), or through untutored learning experiences (e.g., Green, 2002; Veblen, 2012).

Classifying someone as musically trained or untrained is not straightforward (Zhang et al., 2020). Here, we considered untrained individuals to be those with *no* formal music lessons—either instrumental or voice. Our focus on formal lessons is consistent with Zhang et al.’s (2020) review of the literature, which concluded that recruitment from music schools and/or 6 years of training represent a consensus for classifying someone as a musician. Others have considered a cut-off of 2 years of lessons to classify participants as musically experienced or inexperienced (e.g., Dowling et al., 1995). For conceptual and theoretical clarity, we opted for a more conservative definition to rule out any potential contribution of formal lessons. This decision left us with the problem of individuals who are clearly musicians even though they have no formal training (e.g., Louis Armstrong, David Bowie). Formal training and untutored learning are two poles of a continuum (Folkestad, 2006; Green, 2002; Veblen, 2012), which typically differ in learning style (formal vs. informal), context (inside institutional settings vs. outside), and goals. Nevertheless, in research on music training, participants without formal music lessons but who practice informally are often included in the same group as participants who never played a musical instrument (e.g., Swaminathan et al., 2017, 2018). Informal practice is typically not even measured. To the best of our knowledge, ours is the first study to examine untutored learning and informal practice in detail.

Because untrained listeners can vary widely in musical ability, due to both genetic factors and informal musical experiences, integrating these differences into studies of musical expertise is bound to be informative. Such integration would be consistent with perspectives on musicality as a broad and multifaceted concept (Müllensiefen et al., 2014). Expanding our understanding of musical abilities beyond the narrow scope of formal music lessons also has implications for the interpretation of findings from studies on music training. For example, if variables typically correlated with training also correlate with musical ability in the absence of training, training would be sufficient but not necessary to explain the advantages observed in musicians. Rather, predispositions and/or informal experiences could influence the development of musical and/or non-musical abilities, *and* the likelihood of taking music lessons. Moreover, if musical abilities and related variables can be as high in subgroups of untrained individuals as in trained musicians, the specificity of training-related differences would be called into question. In short, understanding musicality in the absence of music lessons is essential for a nuanced conceptualisation of musical abilities, and to tease apart training-specific from more general associations.

In the present investigation, we focused on a sample that included only individuals with no formal training in music. Some studies examining correlates of musical ability held music training constant by statistical means (e.g., Kragness et al., 2021; Swaminathan et al., 2017, 2018, 2021) whereas our study held music training constant by selective sampling. Although a previous study examined musically untrained *children* (James et al., 2020), ours is the first to use this approach with adults, who are more likely to have a history of informal music practice. We assessed musical ability objectively using the Musical Ear Test (MET, Wallentin et al., 2010), which has separate subtests for melody and rhythm processing. Participants also completed the Goldsmiths Musical Sophistication Index (Gold-MSI, Müllensiefen et al., 2014), a self-report questionnaire that asks about formal and informal musical behaviours, experience, and skills. We additionally measured participants’ general cognitive abilities and personality traits, two domains often considered in music-training studies (e.g., Kuckelkorn et al., 2021; Swaminathan & Schellenberg, 2018). Finally, we identified untrained listeners from our sample who performed well on the MET, so that we could compare them with trained listeners tested previously but identically by Correia et al. (2022b).

Our main goal was to identify correlates of musical abilities among individuals with no formal music lessons. We were particularly interested in whether cognitive abilities and personality traits that predict years of music lessons (e.g., Corrigall et al., 2013) also predict musical ability among untrained individuals. In samples of individuals who vary widely in music training, musical ability is associated positively with cognitive ability and with the personality trait openness-to-experience (hereafter, *openness;* e.g., Swaminathan et al., 2021; Swaminathan & Schellenberg, 2018). We also asked whether musical ability among untrained individuals would be associated positively with (1) self-reports of musical sophistication measured by the Gold-MSI subscales, and (2) informal music learning and practice measured by specific Gold-MSI items (e.g., number of instruments played, amount of practice). These questions were motivated by previous findings using different objective measures of musical ability, and by the idea that musical ability relates to multiple forms of engagement with music in addition to lessons (Lee & Müllensiefen, 2020; Müllensiefen et al., 2014). Because formal music lessons predict melody skills better than rhythm skills (Correia et al., 2022b; Swaminathan et al., 2021), we also asked whether untutored practice and playing might be differentially associated with the two MET subtests.

A secondary objective was to identify untrained listeners with good musical abilities—so-called *musical sleepers* (Law & Zentner, 2012)—to compare them to trained individuals tested previously by Correia et al. (2022b) in terms of their musical, cognitive, and personality characteristics. We expected that trained individuals, with their years of formal musical experiences, would score higher on the Gold-MSI. Performance on the MET was bound to tell a more interesting story, regardless of the results. If the musical abilities of the best performing untrained listeners fall below those of trained listeners, music training would appear to provide a *unique* pathway for high levels of musicality. Alternatively, if a substantial proportion of untrained participants display levels of musical ability comparable to their trained counterparts, factors other than training (i.e., genetics, informal musical experiences) would be implicated. For measures of cognitive ability and personality, the available literature precluded clear predictions about differences between high-ability untrained participants and trained ones, because ours is the first study to examine these differences, and the first to isolate effects of formal training.

## Method

### Participants

Ethical approval for the study protocol was obtained from the local ethics committee at Iscte-University Institute of Lisbon (reference 07/2021). Informed consent was collected from each participant at the beginning of the experiment. A sample of 861 participants was recruited initially, mainly in response to advertisements posted on social media (e.g., Facebook, LinkedIn), but also via email and snowball sampling. Subsets of this sample were used previously to document the psychometric properties of the online testing format (Correia et al., 2022b, *N* = 608), and to examine how professional musicians differ from other individuals (Vincenzi et al., 2022, *N* = 642).

Because our interest here was in musically untrained individuals, the present sample comprised the 190 individuals (132 women, 58 men) with *no* formal music lessons (instrumental or voice). This criterion was stricter than the one typically used in the literature, in which individuals with up to 2–3 years of lessons are also included in the untrained/nonmusician category (e.g., Anaya et al., 2017; Bidelman et al., 2013; Mankel & Bidelman, 2018). Although our participants had no formal training, 43 answered *yes* when asked if they can play an instrument (or sing), and 27 of these were currently playing (detailed information about musical behaviours other than lessons is provided in Supplementary Table S1).

Additional untrained participants were tested but excluded because of self-reported hearing disabilities (*n* = 2), unspecified gender (*n* = 1), having a music-related job (*n* = 1), or performing significantly *below* chance levels (i.e., scores < 19, chance = 26, normal approximation to the binomial, two-tailed) on either the Melody or Rhythm subtest of the MET (*n* = 32). Such low levels of performance were uninterpretable in terms of musical ability and indicated failing to attend to the task.

Participants ranged in age from 18 to 73 years (median = 27). The average was 32.0 years (*SD* = 16.0). In terms of education, most had a university degree (bachelor’s: *n* = 36, master’s: *n* = 55, Ph.D.: *n* = 14). The rest had completed high school (*n* = 85). Preliminary analyses revealed that performance on MET Melody, Rhythm, and Total Scores improved with increased age, *r*s > .26, *p*s < .001, and education, *r*s > .28, *p*s < .001. Accordingly, age (in years) and education (coded 1-4) were held constant in the analyses that follow. Because men and women scored similarly on the MET, *p*s > .1, gender was not considered further.

To recruit a large and diverse sample, the study was available in four languages (English, Italian, Brazilian Portuguese, and European Portuguese). Our goal was to test as many participants as possible. Post hoc power analyses conducted with G*Power 3.1 (Faul et al., 2007) confirmed that our sample of 190 musically untrained individuals provided power of 80% to detect partial correlations of .20, with two covariates (age, education) held constant. For group comparisons (two covariates), a sub-sample of 51 high-ability untrained participants was compared to 220 trained participants (from Correia et al., 2022b). These samples provided more than 80% power to detect small effect sizes (i.e., partial η^2^ ⩾ 0.03).

The full dataset is available on the OSF platform (https://osf.io/564xy/?view_only=b545f24df7af4a21908c2583032255a7).

### Measures

Gorilla Experiment Builder (Anwyl-Irvine et al., 2020), an online platform for psychological research, was used to adapt questionnaires and tasks, programme the experiment, and collect the data. Original measures were used for the English version of the programme. Published translations for the other languages (Italian, Brazilian Portuguese, European Portuguese) were used when available. When a measure was not validated for a target language, a translated version was created by bilinguals, who were native speakers of the target language and fluent in English. Online versions of all tests had good reliability and validity (Correia et al., 2022), and all are available on Gorilla (https://app.gorilla.sc/openmaterials/218554).

#### Musical expertise

##### Musical Ear Test (MET)

The MET was our objective measure of musical ability (Wallentin et al., 2010). The MET has good reliability and validity, both for in-person (Swaminathan et al., 2021) and online (Correia et al., 2022b) testing. It has two subtests: Melody and Rhythm. On each trial, participants hear a pair of short sequences of piano tones in the Melody subtest, and drumbeats in the Rhythm subtest, and judge whether the two sequences are identical. When the sequences differ, at least one tone (Melody) or one inter-onset interval (Rhythm) is altered. Both subtests include 52 trials (half *identical*) and they are always presented in the same order—Melody then Rhythm—with two initial practice trials for both subtests. Feedback is provided on the practice trials but not on the test trials. Participants have a limited time (1500 ms for Melody, 1659 to 3230 ms for Rhythm) to answer before the presentation of the next trial. Because time intervals between trials are fixed, the MET has the same duration for each participant (20 min; for more details regarding the MET, see Swaminathan et al., 2021).

Before testing began, participants were asked to use headphones and to avoid distractions throughout the test. The number of correct responses was calculated separately for each participant for both subtests and for Total scores. Following the test’s developers (Wallentin et al., 2010), missing responses were considered incorrect.

##### Goldsmiths Musical Sophistication Index (Gold-MSI)

The Gold-MSI is a self-report questionnaire that includes 38 items asking about behaviours, experiences, and skills related to music (Lima et al., 2020; Müllensiefen et al., 2014). For scoring purposes, items are combined to form 5 subscales: Active Engagement (9 items; e.g., *I listen attentively to music for __ per day*), Perceptual Abilities (9 items; e.g., *I can tell when people sing or play out of tune*), Music Training (7 items; e.g., *I have had formal training in music theory for __ years*), Singing Abilities (7 items; e.g., *I am able to hit the right notes when I sing along with a recording*), and Emotions (6 items; e.g., *I often pick certain music to motivate or excite me*). A General Factor score (18 items) is also calculated based on representative items from each subscale. Participants respond on 7-point scales. For most items, they rate their agreement (1 = *completely disagree* to 7 = *completely agree*). For the final seven items, response options vary from item to item. In the example provided above for the Active Engagement subscale, seven response alternatives increase monotonically from *0-15* *min* to *4 hours or more*.

One specific item on the Music Training subscale (*I have had _ years of formal training on a musical instrument [including voice] during my lifetime*) was used to classify participants as musically untrained. Anyone who selected option 1 (i.e., 0 years) was considered untrained. Thus, Music Training subscale scores were not included in the analyses, but the other items from the subscale (except for one about formal training in music theory) remained potentially relevant because they measured experiences that do not require a formal learning context, such as amount of practice and number of musical instruments played.

#### Cognitive abilities

##### General cognitive ability

The Matrix Reasoning Item Bank (MaRs-IB; Chierchia et al., 2019) is an online test of abstract (nonverbal) reasoning similar to Raven’s Advanced Progressive Matrices (Raven, 1965). It has been used successfully in previous studies as a measure of general cognitive ability (hereafter, *cognitive ability*; e.g., Correia et al., 2022b; Nussenbaum et al., 2020). The test includes 80 trials, each comprising a matrix with 9 cells in a 3 x 3 configuration, with each cell containing abstract shapes that vary on one to four dimensions (colour, size, shape, and location). The cell in the bottom-right corner is always empty, and participants choose, from four alternatives, the one that logically completes the matrix.

The MaRs-IB has a duration of 8 min, regardless of the number of responses given by each person. Participants are told in advance that they have a maximum of 30 s to respond to each trial, but they are not informed about the task duration, which means that the number of trials participants complete can vary from 16 to 80. If a participant responds to all the trials in less than 8 min, matrices are re-presented in the same order, but responses from repeated trials are not considered in the final score. Following the scale’s developers (Chierchia et al., 2019), cognitive ability was measured as the proportion of correct responses (i.e., correct responses/number of responses), calculated for each participant after excluding responses given in less than 250 ms. For statistical analyses, proportions were logit-transformed.

##### Mind-Wandering Questionnaire (MWQ)

The MWQ (Mrazek et al., 2013) was included for exploratory purposes, to measure participants’ ability to sustain attention and focus. Because this cognitive ability, like other domain-general ones, is important for many musical activities, we speculated that it would be associated positively with musical ability and experience. The questionnaire includes 5 sentences that represent distinct trait levels of mind-wandering (e.g., *I mind-wander during lectures or presentations*). Participants are asked to evaluate how often each one applies to them, using a 6-point rating scale (1 = *almost never* to 6 = *almost always*). An average score indicates the frequency of mind-wandering, such that *lower* scores are indicative of higher levels of sustained attention and focus.

#### Personality

##### Big-Five Inventory (BFI)

The BFI (John et al., 1991, 2008) is a self-report questionnaire used frequently to measure personality traits from the five-factor model (McCrae & John, 1992): Extraversion, Agreeableness, Conscientiousness, Neuroticism, and Openness-to-Experience. The BFI comprises 44 items, with each item representative of one of the traits (e.g., Extraversion: *I see myself as someone who is talkative*; Agreeableness: *I see myself as someone who likes to cooperate with others*). Using a 5-point rating scale, participants evaluate how much they agree with each expression (1 = *disagree strongly* to 5 = *agree strongly*). A mean score is calculated for each personality trait.

### Procedure

To access the study, participants went online and clicked a hyperlink that led them directly to the *Gorilla* platform (http://www.gorilla.sc/). After they confirmed their willingness to participate and responded to demographic questions (e.g., age, gender, education), they completed one 40 min online session. The questionnaires and tasks were always presented in the same order: the MWQ, Gold-MSI, BFI, MaRs-IB, and finally the MET. The fixed order meant that the objective skills-based tests (MaRs-IB, MET), which were longer in duration, were always at the end of the testing session. After completing all tasks, participants received feedback about their musical abilities and personality. Providing feedback at the end (mentioned during recruitment) was intended to improve motivation to participate and to complete the entire test session.

## Results

### Analysis

In the analyses that follow, we report standard frequentist statistics. Instead of correcting for multiple tests, we also report results from Bayesian analyses using JASP 0.16.1 (JASP Team, 2022) and default priors. Bayesian statistics allowed us to determine whether the observed data were more likely under the null or alternative hypothesis, and whether the evidence was negligible (BF_10_ < 3), substantial (3 < BF_10_ < 10), strong (10 < BF_10_ < 30), very strong (30 < BF_10_ < 100), or decisive (BF_10_ > 100) in this regard (Jarosz & Wiley, 2014; Jeffreys, 1961). Weak but significant results from frequentist statistics were considered unreliable if they were not accompanied by substantial (or stronger) evidence. Bayesian analyses also allowed for a clearer interpretation of null findings when the observed data were substantially more likely (i.e., BF_10_ < .333) under the null than the alternative hypothesis.

The first set of analyses examined individual differences that predict musical ability among participants with no music lessons (age and education held constant). We then identified untrained listeners with good musical abilities (those scoring in the top 25% of the MET Total score range) and asked how they compare to formally trained ones in their musical, cognitive, and personality characteristics. The trained participants were tested previously but identically by Correia et al. (2022b).

### Musically untrained participants

Preliminary analyses confirmed that MET Melody, Rhythm, and Total scores did not vary as a function of the language of the test, *F*s < 1. Test language was not considered further. Descriptive statistics for the MET, Gold-MSI subscales, personality traits from the BFI, and cognitive abilities (MaRs-IB, MWQ) are provided in Supplementary Table S1. The distribution of MET Total scores was unimodal and approximately normal (Shapiro-Wilk test, *p* = .542). The observed data provided very strong evidence that mean levels of performance were lower than those from published norms (72.5; Swaminathan et al., 2021), *t*(189) = 3.54, Cohen’s *d* = .257, BF_10_ = 32.0. This result was expected because the normative sample included individuals who were musically trained.

MET Melody and Rhythm scores were correlated positively, *r* = .579, *N* = 190, *p* < .001, BF_10_ > 100, and the association was similar in magnitude to that reported by Swaminathan et al. (2021; *r* = .489), *z* = 1.71, *p* = .087. Comparisons of correlations from dependent samples were conducted with Psychometrica (https://www.psychometrica.de/correlation.html).

Table 1 reports partial correlations between the MET and the other variables (age and education held constant). Even for our sample of untrained participants, musical ability, as measured by the MET Melody, Rhythm, and Total scores, correlated positively with Gold-MSI scores. The one exception was for the subscale Active Engagement, for which the observed data provided substantial evidence for the *null* hypothesis for Rhythm and Total scores. The association between Melody scores and Active Engagement was negligible, as was the association between Rhythm and Singing Abilities. In all other instances, evidence for a positive association ranged from substantial to decisive. In other words, as performance on our objective measures of musical ability increased, so did self-reports of singing ability, emotional responding to music, perceptual skills, and overall musical sophistication.

**Table 1.** Pairwise correlations between MET scores and Gold-MSI subscales, personality dimensions, cognitive abilities, and mind-wandering (age and education held constant, *N* = 190).

	MET total			MET melody			MET rhythm		
	*r*	*p*	BF10	*r*	*p*	BF10	*r*	*p*	BF10
MET									
Melody	.894	<.001	>100	–	–	–	–	–	–
Rhythm	.883	<.001	>100	.579	<.001	>100	–	–	–
Gold-MSI									
Active Engagement	.045	.535	.261	.068	.351	.340	.011	.880	.237
Perceptual Abilities	.294	<.001	>100	.295	<.001	>100	.227	.002	22.6
Singing Abilities	.230	.002	24.6	.245	<.001	49.1	.161	.027	2.27
Emotion	.270	<.001	>100	.279	<.001	>100	.199	.006	7.54
General Factor	.287	<.001	>100	.305	<.001	>100	.203	.005	8.88
Personality									
Extraversion	–.024	.746	.229	–.051	.484	.285	.011	.885	.236
Agreeableness	.154	.035	1.76	.130	.075	.990	.144	.048	1.43
Conscientiousness	–.029	.691	.235	–.035	.635	.252	–.017	.821	.240
Neuroticism	.036	.621	.245	.022	.769	.236	.043	.554	.275
Openness	.115	.115	.798	.124	.090	.863	.080	.275	.407
Cognition									
Cognitive Ability	.333	<.001	100	.276	<.001	>100	.316	<.001	>100
Mind-Wandering	.076	.303	.359	.068	.356	.337	.067	.364	.343

MET: musical ear test; Gold-MSI: Goldsmiths Musical Sophistication Index.

For personality traits (Table 1), there were no significant correlations between MET scores and Extraversion, Conscientiousness, or Neuroticism, and the data provided substantial evidence for the null hypothesis in each instance. Although Agreeableness was positively correlated with Rhythm and Total scores, the evidence was negligible, as it was for Melody, and for all associations between Openness and MET scores. Finally, performance on the MET had strong positive associations with cognitive ability, with evidence deemed decisive by Bayesian analyses. There were no significant associations with mind wandering, however, although evidence favouring the null hypothesis was negligible. In any event, the results confirmed that among individuals with no music training, musical ability was correlated positively with cognitive ability and with other musical behaviours and experiences.

Table 2 provides partial correlations between the MET and six of the seven individual items from the Gold-MSI Music Training subscale, excluding the item that measured years of formal training on a musical instrument (or voice), which did not vary in our sample. MET scores had no association with formal training in music theory or the degree to which participants identified as musicians, and the observed data provided substantial evidence for the null hypotheses. MET scores correlated positively with the other four items, however, which measured *untutored* music learning and practice. Higher scores on the MET were predicted by years of music practice, daily hours of practice, compliments received about musical ability, and number of instruments played. In all instances, the observed data provided substantial or stronger evidence. Because these four items from the Gold-MSI were intercorrelated, *r*s ⩾ .388, *N* = 190. *p*s < .001, we extracted a principal component (hereafter *Music Practice*) to use in subsequent analyses. This latent variable accounted for 67.4% of the variance in the original four items, and each item loaded highly (> .7) onto the latent variable. As shown in Table 2, Music Practice maximised associations with MET scores, although the correlation was significantly higher for the Melody than for the Rhythm subtest, *z* = 2.60, *p* = .009.

**Table 2.** Pairwise correlations between MET scores and individual items from the music training subscale of the Gold-MSI (age and education held constant, *N* = 190).

	MET total			MET melody			MET rhythm		
	*r*	*p*	BF_10_	*r*	*p*	BF_10_	*r*	*p*	BF_10_
Gold-MSI Item									
Duration of Practice	.333	<.001	>100	.355	<.001	>100	.234	.001	30.2
Compliments	.243	<.001	43.7	.257	<.001	86.1	.173	.018	3.17
Identity	.060	.410	.300	.053	.469	.289	.054	.460	.302
Hours of Practice	.331	<.001	>100	.373	<.001	>100	.212	.004	12.3
Music Theory	.052	.478	.276	.060	.411	.310	.032	.667	.255
Instruments Played	.343	<.001	>100	.379	<.001	>100	.227	.002	22.6
Music Practice^a^	.383	<.001	>100	.419	<.001	>100	.258	<.001	92.6

MET: Musical Ear Test; Gold-MSI: Goldsmiths Musical Sophistication Index.

a Principal component extracted from the other items (except Music Theory and Identity).

Because our measure of Music Practice was novel, we asked whether it was associated with individual differences in openness and cognitive ability, as music training is. The observed data provided very strong evidence that Music Practice was associated positively with openness, *r* = .238, *p* < .001, BF_10_ = 38.5, but there was no association with cognitive ability, *r* = .118, *p* = .105, BF_10_ = .937, although evidence for the null hypothesis was negligible. In short, individuals who were high in openness had an increased likelihood of informal music practice.

In the analyses, we used multiple regression to determine which combination of variables best predicted MET scores. The model included age, education, the Gold-MSI General Factor (to reduce collinearity), Music Practice, and cognitive ability. Results are provided in Table 3. The model was significant in each case, with age and cognitive ability making significant independent contributions in each instance, and Music Practice making a significant independent contribution for Melody and Total scores, but not for Rhythm scores. For all significant partial associations, Bayesian analyses confirmed that the observed data provided strong to decisive evidence. For the association between Music Practice and Rhythm scores, Bayesian analyses indicated that the observed data were equally likely under the null and alternative hypotheses. As before, the partial association between Music Practice and Melody scores (*r* = .272) was stronger than the partial association between Music Practice and Rhythm Scores (*r* = .125), *z* = 2.09, *p* = .037.

**Table 3.** Multiple regression results predicting MET scores from age, education, the Gold-MSI general factor, music practice, and cognitive ability.

	MET total			MET melody			MET rhythm		
	*R* ^2^	*p*	BF_10_	*R* ^2^	*p*	BF_10_	*R* ^2^	*p*	BF_10_
Model	.337	<.001	>100	.319	<.001	>100	.239	<.001	>100
	ß	*p*	BF_10_	ß	*p*	BF_10_	ß	*p*	BF_10_
Predictors									
Age	.314	<.001	>100	.286	<.001	>100	.280	<.001	78.2
Education	.142	.054	1.17	.146	<.052	1.25	.110	.165	.585
Gold-MSI	.098	.228	.396	.081	.328	.321	.097	.268	.419
Music Practice	.261	.002	23.4	.316	<.001	>100	.149	.090	.915
Cognitive Ability	.299	<.001	>100	.238	<.001	84.5	.303	<.001	>100

MET: Musical Ear Test; Gold-MSI: Goldsmiths Musical Sophistication Index.

### Comparison of musically untrained and trained individuals

**Figure 1.**
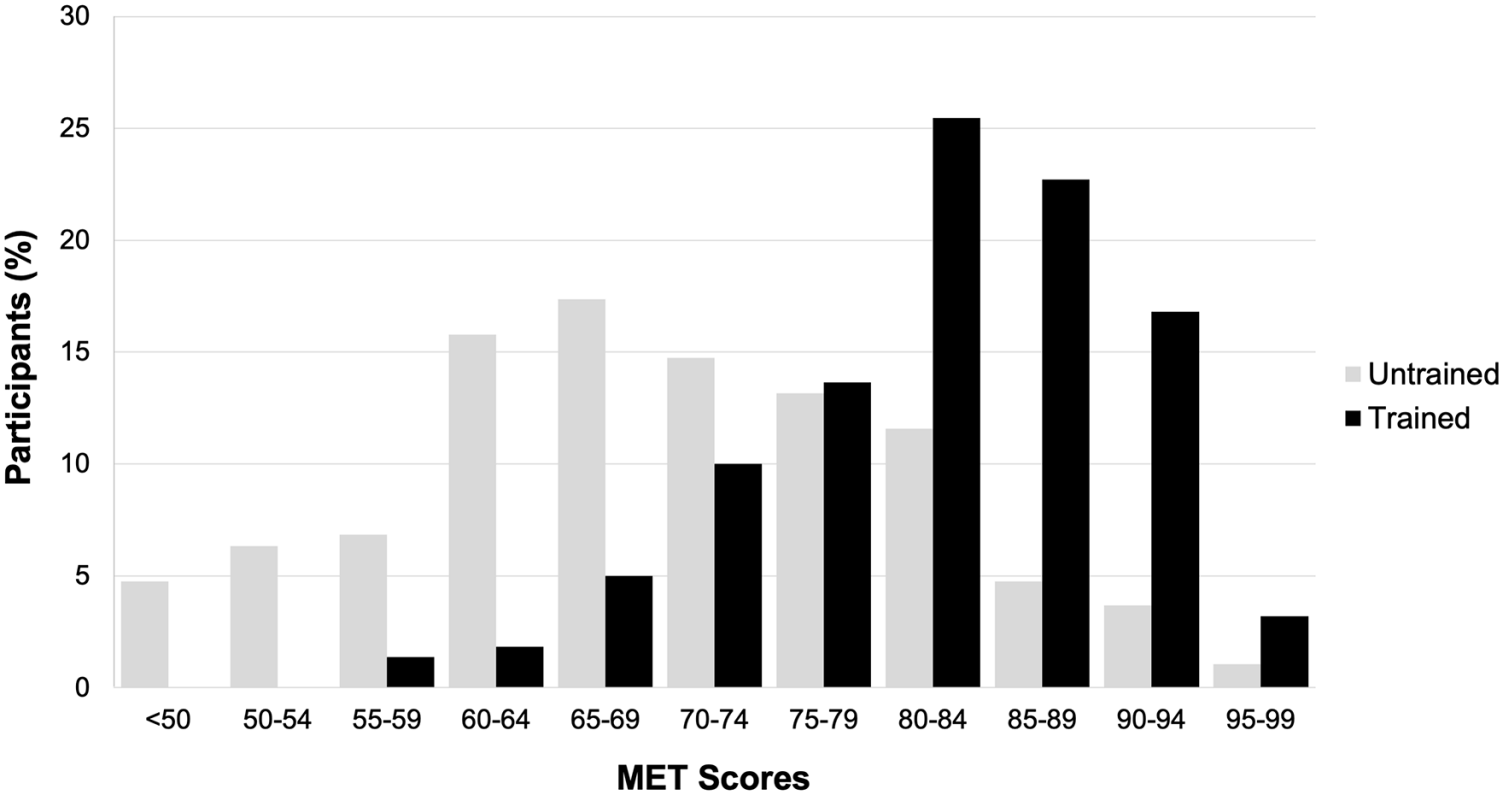
Distribution of MET total scores for untrained and trained participants.

We then compared our untrained participants with the 220 musically trained ones from Correia et al. (2022b), each of whom had at least 6 years of lessons, as per the criterion used in most music-training research (Zhang et al., 2020). No trained individual had a Melody or Rhythm score that was significantly below chance levels. Figure 1 illustrates descriptive statistics for MET Total scores separately for the two groups. An Analysis of Covariance with music training as a between-subjects variable and two covariates (age, education) confirmed that Total scores for trained individuals were decisively higher than those for untrained individuals, *F*(1, 403) = 134.69, *p* < .001, partial η^2^ = .250, BF_10_ > 100. Nevertheless, the distributions overlapped considerably. In fact, 12% of the untrained individuals (*n* = 23) scored above the mean (82.2) and median (82.5) for the trained individuals. The figure also shows considerable variation in MET Total scores for both groups, although scores varied more for the untrained compared to the trained participants. *F*(1, 405) = 14.04, *p* < .001 (Levene’s test for equality of variances).

The overlap between distributions motivated us to ask if musically untrained individuals with high levels of ability are similar to trained individuals in terms of musical abilities, cognitive abilities, and personality. To avoid focusing on particularly unusual or extreme cases, we selected untrained individuals who had MET Total scores in the top 25% (i.e., MET Total score ⩾ 78 out of 104; *n* = 51).

Compared to the trained individuals from Correia et al. (2022b), the high-ability untrained participants did not differ in age, education, or gender, *p*s > .09. There was decisive evidence, however, that the trained individuals were more likely to play a musical instrument (or sing), χ^2^(1, *N* = 271) = 112.04, *p* < .001, ϕ = .643, BF_10_ > 100 (trained: 218/220, untrained: 25/51), and to be currently playing music, χ^2^(1, *N* = 271) = 52.23, *p* < .001, ϕ = .439, BF_10_ > 100 (trained: 177/220, untrained: 15/51).

As shown in Table 4, high-ability untrained participants had MET Total scores similar to those of the trained participants, although evidence for the null hypothesis was negligible. The groups also did not differ on the Melody subtest, with substantial evidence favouring the null hypothesis. There was strong evidence, however, that *untrained* participants had *higher* Rhythm scores, which, in turn, led to strong evidence for an interaction between group and subtest, *F*(1, 264) = 11.45, *p* < .001, partial η^2^ = .042, BF_10_ = 17.7.

**Table 4.** Descriptive statistics for high-ability musically untrained participants (top 25%) and trained participants from Correia et al. (2022). Age and education were held constant in statistical comparisons.

	High-ability untrained (*n* = 51)	Trained (*n* = 220)				
	*M* (*SD*)	*M* (*SD*)	*F*	*p*	BF_10_	Partial η^2^
MET						
Total	83.9 (5.2)	82.0 (8.3)	1.88	.171	.407	.007
Melody	41.5 (3.9)	41.9 (3.9)	<1	.484	.206	.002
Rhythm	42.5 (3.2)	40.2 (4.5)	10.99	.001	27.5	.040
Gold-MSI						
Active Engagement	3.9 (1.3)	5.0 (0.9)	48.51	<.001	>100	.155
Perceptual Abilities	5.1 (1.1)	6.2 (0.6)	82.11	<.001	>100	.237
Singing Abilities	4.0 (1.6)	5.2 (0.9)	53.26	<.001	>100	.168
Emotion	5.6 (1.0)	6.0 (0.7)	8.13	.005	7.39	.030
General Factor	3.7 (1.2)	5.5 (0.7)	193.70	<.001	>100	.423
Music Practice	–1.6 (1.1)	0.4 (0.5)	334.70	<.001	>100	.559
Personality						
Extraversion	3.3 (0.9)	3.3 (0.8)	<1	.888	.169	<.001
Agreeableness	3.9 (0.4)	3.9 (0.5)	<1	.715	.179	<.001
Conscientiousness	3.7 (0.7)	3.7 (0.7)	<1	.399	.232	.003
Neuroticism	3.0 (0.8)	3.0 (0.9)	<1	.629	.183	<.001
Openness	3.9 (0.6)	4.2 (0.5)	21.76	<.001	>100	.076
Cognition						
Cognitive Ability	0.7 (0.8)	0.6 (0.7)	1.00	.318	.270	.004
Mind-Wandering	3.2 (1.1)	3.0 (0.9)	4.76	.030	1.48	.018

MET: musical ear test; Gold-MSI: Goldsmiths Musical Sophistication Index.

For self-reports of musical sophistication (i.e., the subscales and general factor of the Gold-MSI), trained participants scored consistently higher than their untrained but high-ability counterparts. In fact, the observed data provided decisive evidence for a group difference on all subscales except Emotions, for which the evidence remained substantial. When we re-extracted the principal component (i.e., Music Practice, 63.2% of variance explained) using the same four items from the Gold-MSI Music Training subscale (excluding years of music lessons, music theory, and musical identity), musically trained individuals had decisively higher scores on this latent variable.

For personality traits, the trained group had decisively higher scores on openness, but not on any other personality trait, for which the observed data provided consistent and substantial support for *null* associations. There was also substantial evidence that the groups did not differ in cognitive ability. Finally, although the trained group had significantly lower mind-wandering scores, the evidence was negligible in this regard.

These findings did not change when we compared trained individuals to untrained individuals who scored in the top 20% (*n* = 40) or 30% (*n* = 58) for MET Total scores. Results are summarised in Supplementary Tables S2 and S3. Specifically, the untrained group scored higher on Rhythm scores, there was an interaction between MET subtest and group, the trained group had higher openness scores, and the trained group had higher scores on all Gold-MSI subscales, the general factor, and the latent Music Practice variable.

Finally, to isolate further the role of formal music lessons, we compared our high-ability untrained participants to trained participants who had equally high MET Total scores (⩾78, *n* = 163). Results are provided in Supplementary Table S4. The two high-ability groups did not differ in age, education, or gender, *p*s > .2, but there was decisive evidence that the trained participants were more likely to play a musical instrument (or sing), χ^2^(1, *N* = 214) = 89.38, *p* < .001, ϕ = .643, BF_10_ > 100 (trained: 162/163, untrained: 25/51), and to be currently playing, χ^2^(1, *N* = 214) = 51.20, *p* < .001, ϕ = .489, BF_10_ > 100 (trained: 134/163, untrained: 15/51). The trained group had substantially higher MET total scores, which stemmed from a decisive advantage on the Melody subtest. The former advantage for untrained participants on the Rhythm subtest became non-significant, although evidence for a null association was negligible. Nevertheless, the interaction between group and subtest remained decisive, *F*(1, 208) = 18.42, *p* < .001, partial η^2^ = .081, BF_10_ > 100. The results remained unchanged for the other individual-difference variables (Gold-MSI, personality, and cognitive abilities).

## Discussion

Variables that predicted musical abilities among musically untrained individuals included higher levels of cognitive ability and self-reported musical experiences and skills, particularly untutored music practice and playing. Untrained participants varied widely in musical abilities, however, and there was substantial overlap in the distribution of trained and untrained participants (Figure 1). In fact, many untrained participants (12%) had better musical abilities than the average trained participant. Moreover, untrained participants with particularly good musical abilities (MET scores in the top 25%) were comparable to trained musicians in cognitive ability and melody processing, and better in rhythm processing. They were lower, however, in the personality trait openness.

Our results from the top untrained performers (regarding musical and cognitive ability) are consistent with evidence of genetic contributions to musical ability and achievement (Hambrick & Tucker-Drob, 2015; Mosing et al., 2014; Tan et al., 2014; Wesseldijk et al., 2019), and with results from studies of nonmusicians reporting positive associations between musicality and nonmusical abilities (Correia et al., 2022a; Gingras et al., 2015; Mankel & Bidelman, 2018; Morrill et al., 2015; Swaminathan & Schellenberg, 2017). In other words, some musical and nonmusical differences between trained and untrained individuals do not appear to be the sole consequence of formal music lessons, a finding that is relevant to contentious debates about music training and plasticity (Bigand & Tillmann, 2022; Sala & Gobet, 2020). This finding also highlights the importance of measuring musical abilities *and* music training to tease apart training-specific from more general associations.

Our finding that cognitive ability predicted musical abilities in the absence of formal training extends previous results from individuals who varied widely in training (e.g., Swaminathan et al., 2017, 2018; Swaminathan & Schellenberg, 2018, 2020). Indeed, the magnitude of the association between cognitive and musical *abilities* that we observed was comparable to associations that have been reported between cognitive ability and music training (e.g., Degé et al., 2011; Schellenberg, 2006; Swaminathan & Schellenberg, 2018). Perhaps listeners with higher cognitive ability perform better on virtually any test (Carroll, 1993), including music-discrimination tasks such as the MET, which makes them better able to deal with the demands of musical activities and more likely to pursue music training (Mosing et al., 2019). By contrast, and unexpectedly, there was no association between musical ability and openness, even though openness predicts musical ability in studies of musicians (Butkovic et al., 2015; Kuckelkorn et al., 2021; Vincenzi et al., 2022) and individuals who vary in music training (Corrigall et al., 2013; Thomas et al., 2016). Nevertheless, the association between openness and our Music Practice variable suggests that open individuals are more likely to practice and play music actively, whether or not formal training is involved.

Observed associations between musical ability and the Gold-MSI subscales, and between musical ability and untutored Music Practice, highlight the multifaceted nature of musicality. These associations do not appear to be task-specific, because they extend to other ways of measuring musical ability using objective tests and self-reports (Kunert et al., 2016; Law & Zentner, 2012; Lee & Müllensiefen, 2020; Müllensiefen et al., 2014). One possibility is that individual differences in musical behaviours determine musical ability, including low-level discrimination skills. Alternatively, pre-existing levels of musical ability could influence musical behaviours and levels of engagement with music, or a third unidentified variable could be involved. In our view, however, it is more likely that individuals with higher levels of musical ability have an increased probability of practising music informally and engaging with music in various ways, which in turn enhances their ability further—a classic gene-environment correlation, which Scarr and McCartney (1983) called *niche-picking*.

Untutored music practice proved to be a better predictor of performance on the Melody compared to the Rhythm subtest. Other studies that used the MET reported a similar finding with formal music training, which was a better predictor of Melody than of Rhythm (e.g., Swaminathan et al., 2021; Wallentin et al., 2010). In a study of adults (Thomas et al., 2016, Table 1) that used a different music-training variable (number of music classes), training had a stronger association with Melody than with Rhythm scores. Similarly, in a study of children (Ilari et al., 2016), a 1-year music programme led to greater improvements in the children’s ability to discriminate melodies than rhythms. For our sample of untrained participants, however, performance on the Melody and Rhythm subtests was *not* associated with scores on the Active Engagement subscale from the Gold-MSI, which indexes behaviours such as searching the internet for music-related items, commenting about music in posts on social media, and time spent listening attentively to music. In short, strong associations with Melody scores appear to be limited to *active* music playing and practice, regardless of tutoring, learning context, and the player’s goals. Perhaps melody processing is more amenable to learning, whereas rhythm is more stable. Swaminathan et al. (2021) speculated that this might be the reason why rhythm is present in the music of all cultures, but melody is not. It is also possible that specific aspects of informal music practice promote melody processing, such as choosing to play the violin rather than the drums.

On the one hand, then, informal music practice among our untrained participants was linked more strongly to performance on the Melody than the Rhythm subtest. On the other hand, high levels of overall musical ability (i.e., MET Total scores) were a consequence of particularly high *Rhythm* scores. In fact, high-ability untrained participants performed similarly to the average trained participant on the Melody subtest, but higher on the Rhythm subtest. When the comparison was restricted to equally high-ability trained participants, the two-way interaction between group and subtest remained strong, with the trained group performing better on Melody, but no group difference on Rhythm. As in Swaminathan et al. (2021), moreover, performance on the Rhythm subtest was more closely linked to cognitive ability. Other findings show that rhythm abilities predict language abilities (Gordon et al., 2015; Lee et al., 2020; Swaminathan & Schellenberg, 2017, 2020), and that they are better than melody abilities at predicting *future* musical abilities in general—not just rhythm processing (Kragness et al., 2021). Compared to melody processing, then, rhythm may represent a more fundamental musical ability, which helps to explain further its universality as well as its stability.

As one might expect, our untrained participants—even those with high MET scores—were less likely to play a musical instrument and had lower levels of current music practice compared to trained participants. The untrained group also had lower levels of other musical experiences and skills, as measured by the Gold-MSI. Higher scores on all music-behaviour variables were expected because participants with several years of music training would be more likely to engage regularly with a variety of musical activities.

The main limitation of our findings is that we used a single, relatively low-level measure of musical ability, with only two subtests. Thus, our results may not generalise to other tests of musical ability that have additional subtests (Law & Zentner, 2012; Ullén et al., 2014; Zentner & Strauss, 2017). Although the MET has been used widely and correlates with other measures of musical expertise and with music training (e.g., Hansen et al., 2013; Slevc et al., 2016; Swaminathan et al., 2021; Wallentin et al., 2010), future studies could use alternative tests of musical ability, as well as measures that evaluate lower-level abilities such as sound segregation and frequency or temporal discrimination. In addition, the MET considers missing responses to be incorrect, which could lower scores and/or add noise to the data, particularly in an online study. Nevertheless, missing responses are considered incorrect on many psychological tests with forced-choice judgements, including other tests of musical ability (e.g., Peretz et al., 2003; Ullén et al., 2014; Vuvan et al., 2018), as well as tests of general cognitive ability (e.g., Raven, 1965). Moreover, when Correia et al. (2022b) excluded participants with consecutive missing responses on the MET, the test’s psychometric properties were not affected negatively.

In our sample, increases in age predicted improved performance on the MET (Table 3). Although a pattern of decline could be expected based on the cognitive ageing literature (e.g., Grady, 2012; Salthouse, 2019), age-related trajectories in music perception are not necessarily characterised by a decline (Halpern, 2020). In any event, our sample was less than ideal for testing ageing effects (only 23 participants were over 40 years old, and only 8 over 65). We speculate that the positive association with age stems from cumulative exposure to music. Alternatively, many of our younger participants were undergraduate students, who perhaps had less motivation to score well on the MET, compared to older participants who were recruited primarily from the community.

To conclude, the present study provided evidence that predictor variables typically associated with music training also predict musical ability in the absence of training, except for the personality trait openness, which predicted informal music practice but not musical ability. The association between *informal* music practice and performance on the Melody subtest was strong, which implies that such practice should be considered when studying untrained individuals. Regardless, our results confirm that formal music lessons are *not* required to develop good musical abilities, or for associations between musical and nonmusical domains to emerge. Different pathways, namely informal engagement with music and genetic predispositions, appear to play an important role, although many hours of deliberate practice are obviously essential for skilled performance (Ericsson, 2008). In our view, the musicality of untrained participants needs to be considered seriously to develop a complete understanding of associations between music training and nonmusical abilities. Musical expertise and musical ability are more than just taking music lessons.

## Supplemental Material

sj-docx-1-qjp-10.1177_17470218221128557 – Supplemental material for Individual differences in musical ability among adults with no music training Click here for additional data file. Supplemental material, sj-docx-1-qjp-10.1177_17470218221128557 for Individual differences in musical ability among adults with no music training by Ana Isabel Correia, Margherita Vincenzi, Patrícia Vanzella, Ana P Pinheiro, E Glenn Schellenberg and Císar F Lima in Quarterly Journal of Experimental Psychology
