# Supplementary material for: Individual differences in musical ability among adults with no music training
Source: Q J Exp Psychol (Hove). 2022 Oct 27;76(7):1585–98. doi: 10.1177/17470218221128557 (PMC10280665; doi:10.1177/17470218221128557)
Supplement: sj-docx-1-qjp-10.1177_17470218221128557 – Supplemental material for Individual differences in musical ability among adults with no music training [file sj-docx-1-qjp-10.1177_17470218221128557.docx]

**Supplementary Materials for “Individual Differences in Musical Ability Among Adults with No Music Training”**

**Table S1**

*Descriptive Statistics for the MET, Gold-MSI Subscales, Personality Dimensions, Cognitive Abilities, and Mind-Wandering (*N *= 190).*

|  |  | *M* | *SD* | Range of Responses |
| --- | --- | --- | --- | --- |
| **MET** |  |  |  |  |
| Total |  | 69.52 | 11.62 | 42 – 98 |
| Melody |  | 33.83 | 6.62 | 21 – 51 |
| Rhythm |  | 35.68 | 6.27 | 20 – 48 |
| **Gold-MSI** |  |  |  |  |
| Active Engagement |  | 3.85 | 1.22 | 1.22 – 6.67 |
| Perceptual Abilities |  | 4.78 | 1.09 | 1.78 – 7.00 |
| Singing Abilities |  | 3.55 | 1.33 | 1.00 – 6.57 |
| Emotion |  | 5.45 | 0.94 | 2.33 – 7.00 |
| General Factor |  | 3.30 | 1.01 | 1.11 – 5.61 |
| Music Practice |  | 0.00 | 1.00 | -0.77 – 3.53 |
| *Duration of Practice** |  | 1.76 | 1.55 | 1 – 7 |
| *Compliments** |  | 3.28 | 2.28 | 1 – 7 |
| *Identity* |  | 1.74 | 1.34 | 1 – 7 |
| *Hours of Practice** |  | 1.88 | 1.56 | 1 – 7 |
| *Music Theory* |  | 1.46 | 1.25 | 1 – 7 |
| *Instruments Played** |  | 1.42 | 0.74 | 1 – 4 |
| **Personality** |  |  |  |  |
| Extraversion |  | 3.29 | 0.80 | 1.25 – 5.00 |
| Agreeableness |  | 3.73 | 0.53 | 2.22 – 4.89 |
| Conscientiousness |  | 3.53 | 0.69 | 1.67 – 5.00 |
| Neuroticism |  | 3.14 | 0.84 | 1.00 – 5.00 |
| Openness |  | 3.77 | 0.58 | 1.70 – 5.00 |
| **Cognition** |  |  |  |  |
| Cognitive Ability |  | 0.61 | 0.15 | 0.25 – 0.95 |
| Mind Wandering |  | 3.33 | 0.93 | 1.20 – 5.80 |
| Individual items from the Music Training subtest of the Gold-MSI (except Years of Instrumental Lessons) are in italics. *Items used to extract the principal component—Music Practice. | | | | |

**Table S2**

*Descriptive Statistics for High-Ability Musically Untrained Participants (Top 20%) and Trained Participants from Correia et al. (2022). Age and Education Were Held Constant in Statistical Comparisons.*

|  |  | High-Ability Untrained  (*n* = 40) | Trained  (*n* = 220) |  |  |  |  |
| --- | --- | --- | --- | --- | --- | --- | --- |
|  |  | *M* (*SD*) | *M* (*SD*) | *F* | *p* | BF_10_ | η^2^ |
| **MET** |  |  |  |  |  |  |  |
| Total |  | 85.4 (5.0) | 82.0 (8.3) | 5.37 | .021 | 2.12 | .020 |
| Melody |  | 42.2 (4.0) | 42.2 (4.0) | < 1 | .848 | 0.70 | <.001 |
| Rhythm |  | 43.2 (3.1) | 40.2 (4.5) | 15.73 | <.001 | >100 | .057 |
| **Gold-MSI** |  |  |  |  |  |  |  |
| Active Engagement |  | 3.9 (1.2) | 5.0 (0.9) | 39.63 | <.001 | >100 | .134 |
| Perceptual Abilities |  | 5.2 (1.1) | 6.2 (0.6) | 67.87 | <.001 | >100 | .208 |
| Singing Abilities |  | 4.1 (1.5) | 5.2 (0.9) | 36.5 | <.001 | >100 | .124 |
| Emotion |  | 5.7 (1.0) | 6.0 (0.7) | 5.93 | .016 | 2.84 | .022 |
| General Factor |  | 3.8 (1.2) | 5.5 (0.7) | 146.4 | <.001 | >100 | .364 |
| Music Practice |  | -0.6 (0.8) | 0.8 (0.4) | 254.1 | <.001 | >100 | .498 |
| **Personality** |  |  |  |  |  |  |  |
| Extraversion |  | 3.3 (0.9) | 3.3 (0.8) | < 1 | .918 | 0.19 | <.001 |
| Agreeableness |  | 3.9 (0.5) | 3.9 (0.5) | < 1 | .611 | 0.21 | .001 |
| Conscientiousness |  | 3.7 (0.8) | 3.7 (0.7) | < 1 | .479 | 0.24 | .002 |
| Neuroticism |  | 3.1 (0.8) | 3.0 (0.9) | 1.39 | .240 | 0.34 | .005 |
| Openness |  | 3.8 (0.6) | 4.2 (0.5) | 24.97 | <.001 | >100 | .087 |
| **Cognition** |  |  |  |  |  |  |  |
| Cognitive Ability |  | 0.7 (0.8) | 0.6 (0.7) | < 1 | .399 | 0.25 | .002 |
| Mind Wandering |  | 3.1 (1.0) | 3.0 (0.9) | 3.09 | .080 | 0.75 | .011 |

**Table S3**

*Descriptive Statistics for High-Ability Musically Untrained Participants (Top 30%) and Trained Participants from Correia et al. (2022). Age and Education Were Held Constant in Statistical Comparisons.*

|  |  | High-Ability Untrained  (*n* = 58) | Trained  (*n* = 220) |  |  |  |  |
| --- | --- | --- | --- | --- | --- | --- | --- |
|  |  | *M* (*SD*) | *M* (*SD*) | *F* | *p* | BF_10_ | η^2^ |
| **MET** |  |  |  |  |  |  |  |
| Total |  | 83.0 (5.5) | 82.0 (8.3) | < 1 | .502 | .191 | .002 |
| Melody |  | 40.9 (4.1) | 41.9 (5.3) | 2.31 | .130 | .459 | .008 |
| Rhythm |  | 42.1 (3.3) | 40.2 (4.5) | 9.16 | .003 | 12.3 | .033 |
| **Gold-MSI** |  |  |  |  |  |  |  |
| Active Engagement |  | 3.8 (1.3) | 5.0 (0.9) | 57.91 | <.001 | >100 | .176 |
| Perceptual Abilities |  | 5.1 (1.1) | 6.2 (0.6) | 95.78 | <.001 | >100 | .261 |
| Singing Abilities |  | 3.9 (1.5) | 5.2 (0.9) | 64.83 | <.001 | >100 | .193 |
| Emotion |  | 5.6 (0.9) | 6.0 (0.7) | 8.77 | .003 | 9.66 | .031 |
| General Factor |  | 3.6 (1.2) | 5.5 (0.7) | 232.45 | <.001 | >100 | .462 |
| Music Practice |  | -0.7 (0.8) | 0.8 (0.4) | 371.69 | <.001 | >100 | .578 |
| **Personality** |  |  |  |  |  |  |  |
| Extraversion |  | 3.4 (0.8) | 3.3 (0.8) | < 1 | .448 | .215 | .002 |
| Agreeableness |  | 3.9 (0.5) | 3.9 (0.5) | < 1 | .819 | .165 | <.001 |
| Conscientiousness |  | 3.6 (0.7) | 3.7 (0.7) | 1.65 | .200 | .336 | .006 |
| Neuroticism |  | 3.0 (0.8) | 3.0 (0.9) | < 1 | .249 | .180 | <.001 |
| Openness |  | 3.9 (0.6) | 4.2 (0.5) | 22.94 | <.001 | >100 | .078 |
| **Cognition** |  |  |  |  |  |  |  |
| Cognitive Ability |  | 0.7 (0.8) | 0.6 (0.7) | 1.23 | .268 | .274 | .005 |
| Mind Wandering |  | 3. (1.0) | 3.0 (0.9) | 5.88 | .016 | 2.29 | .021 |

**Table S4**

*Descriptive Statistics for High-Ability Musically Untrained Participants (Top 25%) and High-Ability Trained Participants from Correia et al. (2022). Age and Education Were Held Constant in Statistical Comparisons.*

|  |  | High-Ability Untrained  (*n* = 51) | Trained  (*n* = 163) |  |  |  |  |
| --- | --- | --- | --- | --- | --- | --- | --- |
|  |  | *M* (*SD*) | *M* (*SD*) | *F* | *p* | BF_10_ | Partial η^2^ |
| **MET** |  |  |  |  |  |  |  |
| Total |  | 83.9 (5.2) | 86.0 (4.8) | 6.89 | .009 | 4.10 | .032 |
| Melody |  | 41.5 (3.9) | 44.0 (3.6) | 19.90 | <.001 | >100 | .087 |
| Rhythm |  | 42.5 (3.2) | 41.9 (3.0) | 1.36 | .244 | .325 | .007 |
| **Gold-MSI** |  |  |  |  |  |  |  |
| Active Engagement |  | 3.9 (1.3) | 5.0 (0.9) | 47.11 | <.001 | >100 | .185 |
| Perceptual Abilities |  | 5.1 (1.1) | 6.3 (0.6) | 80.36 | <.001 | >100 | .279 |
| Singing Abilities |  | 4.0 (1.6) | 5.3 (0.9) | 56.73 | <.001 | >100 | .214 |
| Emotion |  | 5.6 (1.0) | 6.0 (0.7) | 8.12 | .005 | 7.18 | .038 |
| General Factor |  | 3.7 (1.2) | 5.5 (0.7) | 196.42 | <.001 | >100 | .486 |
| Music Practice |  | -1.6 (1.1) | 0.4 (0.5) | 291.55 | <.001 | >100 | .584 |
| **Personality** |  |  |  |  |  |  |  |
| Extraversion |  | 3.3 (0.9) | 3.4 (0.9) | < 1 | .689 | .188 | <.001 |
| Agreeableness |  | 3.9 (0.4) | 3.9 (0.6) | < 1 | .791 | .176 | <.001 |
| Conscientiousness |  | 3.7 (0.7) | 3.7 (0.7) | < 1 | .329 | .259 | .005 |
| Neuroticism |  | 3.0 (0.8) | 2.9 (0.9) | < 1 | .594 | .201 | .001 |
| Openness |  | 3.9 (0.6) | 4.2 (0.5) | 19.46 | <.001 | >100 | .086 |
| **Cognition** |  |  |  |  |  |  |  |
| Cognitive Ability |  | 0.7 (0.8) | 0.7 (0.7) | < 1 | .862 | .185 | <.001 |
| Mind Wandering |  | 3.2 (1.1) | 2.9 (0.9) | 4.95 | .027 | 1.57 | .023 |
